# Unpacking the Impact and Experience of Working With Lived Experience Partners in Mental Health Research—Exploring Research Team and Public Partner Perspectives

**DOI:** 10.1111/hex.70925

**Published:** 2026-10-16

**Authors:** Joanna Fox, Roya Kamvar, Vanessa Pinfold, Annabel Walsh, Polly‐Anna Ashford, Rosie Lindsay, Jesus Perez, Peter B. Jones, Debra Russo

**Affiliations:** ^1^ Anglia Ruskin University Cambridge UK; ^2^ The McPin Foundation London UK; ^3^ Norwich Clinical Trials Unit, Norwich Medical School University of East Anglia Norwich UK; ^4^ THIS Institute University of Cambridge Cambridge UK; ^5^ CAMEO, Cambridgeshire & Peterborough NHS Foundation Trust Cambridge UK; ^6^ Department of Psychiatry University of Cambridge Cambridge UK

## Abstract

**Introduction:**

Patient and Public Involvement (PPI) in mental health research is encouraged by funders in the United Kingdom, supported by guidance and best practice standards. This includes a commitment to learning and understanding impact. This paper explores the experiences of individuals who worked together for 7 years within an intervention study to understand differing perspectives and conceptualisations of how PPI shaped the research study.

**Methods:**

Using an instrumental case study design, qualitative data were collected in three workshops: university‐based study team (*n* = 4), Lived Experience Advisory Panel (LEAP) (*n* = 5), and mixed composition (*n* = 8). Two workshops explored study set up and intervention development, the other explored a qualitative process evaluation study within the randomised controlled trial.

**Results:**

We recognised impact as tangible and practical, both facilitated and hindered through relational connections which contribute to ways of working that frame research. Data evidenced that both LEAP and study team members experienced a journey of learning and development in doing PPI that included friction and ‘choppy waters’. The study team valued lived experience partners and the knowledge and expertise they brought from the outset. However, issues such as low confidence in doing PPI among study team, changes in PPI team structure, COVID‐19 pandemic moving work online and challenging some LEAP members over the relevance of their own lived experiences to the study, meant the journey was at times difficult.

**Discussion:**

Impact is about much more than quantifiable economic value. It is nuanced and benefits from multi‐perspective explorations that move beyond surface‐level practical observations to achieve deeper understanding of how lived experience expertise shapes complex research processes, across extended time periods, including the role of team dynamics.

**Conclusion:**

PPI in research requires flexibility and open‐mindedness to create a positive inclusive team culture and benefits from regular reflective learning with open feedback, so that timely tangible changes can be agreed.

**Public Contributions:**

This study was designed by LEAP members and the PPI study coordinator employed at a mental health charity. Workshops were co‐facilitated by two people working from a lived experience perspective who are lead authors on this paper.

**Trial Registration**: ISRCTN93895792.

## 1 Introduction

Patient and Public Involvement (PPI) in health and social care research has become an increasing part of the UK research landscape over the last 20 years [1, 2]. Early studies including in the field of mental health [3, 4] have contributed to the development of emancipatory methodologies for knowledge co‐creation including survivor research [5], peer research [6] and PPI [7]. The involvement of lived experience partners is now mandated by major funders in the United Kingdom, including Wellcome Trust, NIHR and UKRI. It has not, however, advanced uncritically with PPI challenged for undermining survivor knowledge and problems of tokenism [8], for being conceptually vague [9] and providing insufficient opportunities for lived experience research leadership [10]. The NIHR defines public involvement in research ‘as research being carried out ‘with’ or ‘by’ members of the public rather than ‘to’, ‘about’ or ‘for’ them. It is an active partnership between patients, carers, members of the public and researchers that influences and shapes research [11]. At the heart of PPI are relationships, including with clinicians, clinical academics, and university‐based researchers who are embedded within hierarchical power‐based systems [12]. When the lived experience knowledge base interacts with clinical systems, there can be challenges; examples are found from lived experience roles within services such as peer support [13] as well as research and academic positions [14].

Until recently, the rationale for much PPI focused on democratic principles; it was seen as the ‘right thing to do’ because PPI increased the relevance of research to people with lived experience [15]. More recently, there has been a greater awareness of epistemic injustice and knowledge co‐creation processes [16]. Supporting guidance on PPI to date has often focused on process, such as challenges in developing collaborative partnerships [17], how to work with PPI research advisory groups [18], how PPI increases patient empowerment [19] and how to report PPI in publications [20]. A 2012 systematic review called for the building of a stronger evidence base to ensure the full impact of PPI could be understood [21]. Exploring questions of worth, impact, and concomitant benefits counter‐balanced with concerns about PPI validity and value for money.

Unpacking the question of impact is not straightforward. How do you measure an embedded process built upon the foundations of personal suffering and guided by principles of meaningful partnership, reciprocity, and power sharing? [22] Impacts of PPI are an underexplored research area but beginning to attract more attention, including reflective case studies [23, 24]. Greenhalgh et al [25] undertook a systematic review of the frameworks to guide PPI and noted that there were over 65 focused on five categories: power‐focused; priority‐setting; study‐focused; report‐focused; and partnership‐focused. This suggests that the purpose of PPI is multi‐faceted and often bespoke to an individual research project; thus, intended impacts will be varied, and research methods must be selected that can explore the heterogeneity of benefits and challenges.

One approach is to learn about and improve models of best practice. Much health research is based on a positivist paradigm, in which impact is a quantifiable element to be measured, without the influence of context [26]. However, studies which have sought to ‘measure’ and ‘assess’ the quantifiable impact of PPI have had mixed results. The NIHR portfolio was explored and those with patient involvement programmes were more likely to reach recruitment targets [27]. A blind RCT exploring patient information sheet accessibility after PPI review found non‐significant results [28]. A systematic review noted the significant contribution that PPI made to patient recruitment in studies, but because of paucity of evidence, it found the impact on retention to be uncertain [29].

Building upon our own experiences of the ‘messy nature’ of collaborative research in practice [30], we set out to understand ‘impact’ experiences in a stepped‐wedge cluster‐randomised controlled trial (RCT) including economic and process evaluation. We do not offer a definition of impact, but explore it by reflecting on the research phases we worked together on. We sought to capture diverse perspectives, not through agreement but in open reflection. What has also been termed dissensus [31]. Thus, our research aim in this paper was to explore the experiences of individuals who worked together for 7 years within an intervention study to understand differing perspectives and conceptualisations of the ‘impact’ of PPI.

### 1.1 Terminology Within the Article

Phrasing used to denote the involvement of people with lived experience in mental health research is disparate, including public contributor, service user researcher, lived experience advisory group member and public partner. In this article, we use both the term public partner and lived experience partner to denote the active involvement of the lived experience perspective throughout the research process.

Lived experience is understood to be personal knowledge about the world gained through direct, first‐hand involvement in everyday events [32]. In this article, the Lived Experience Advisory Panel (LEAP) were people with lived experience of psychosis or of having psychotic experiences, who had used NHS talking therapies during their treatment. Part way through the study a new network—Therapy Involvement Network (TIN)—was formed; these members were formerly study participants and had directly experienced the trial intervention. Members of the TIN were later amalgamated into the LEAP.

## 2 Methodology

A case study design was used to explore PPI impact in the context of this research study [33]. Qualitative data was collected to provide a multi‐perspective understanding from both public partners and the university‐based research study team, exploring how public involvement supported the generation, development and implementation of a clinical trial into NHS Talking Therapies.

### 2.1 Setting

The clinical trial was part of a 7‐year research programme studying a previously unidentified group of people with unmet needs in NHS mental health services [34]. The programme developed and evaluated a complex intervention based on talking therapies to promote recovery. Structurally, the study had all the key PPI components common to NIHR‐funded mental health trials: PPI co‐applicant, LEAP, PPI co‐ordinator post, and an impact log tracking decision making. Additionally, PPI was a standing item on project management agendas as well as weekly team meetings, which were regularly attended by the PPI coordinator.

### 2.2 Study Sample and Recruitment

We co‐produced this project and prioritised the views of current staff and LEAP members who were resourced to work on the study (*n *= 13): 3 male, 10 female; 10 white British/European, 3 ethnic minority communities. In this paper, as in the study, there were overlapping roles; all nine authors were also research participants, and three authors were active facilitators of the data collection process.

Research team composition: Five university‐based team members were involved‐ two academic psychiatrists, one non‐clinical research associate, one clinical research associate and a clinical trial unit manager.

Public partner composition: Eight public partners were involved—four were also researchers with a background in research; one worked at a university as a lecturer. None held clinical roles working in mental health services. LEAP members (*n* = 6) were recruited through open advertisement, with a key criterion being lived experience of psychotic experiences and talking therapies.

Team members consented to take part voluntarily. Some people did not respond to the invite to participate. Everyone interested was given the option to design the study and be an author on outputs. The opportunity to be involved was advertised in team meetings and minutes, but no active follow‐up was actioned to respect people's right not to take part. To ensure participation remained voluntary, participants were offered opt‐outs throughout and a continuous opportunity to discuss any concerns with their team lead privately. Workshops were initially conducted separately to maintain safe spaces to voice perspectives freely amongst working peers.

### 2.3 Data Collection

Data were collected in three daytime, working week, 90‐min long online workshops conducted over MS Teams using a discussion schedule guide. Two workshops explored study set up and intervention development, one the qualitative process evaluation study within the randomised controlled trial. Workshops comprised: University‐based study team (*n* = 4), LEAP (*n* = 5), and mixed team (*n* = 8). The workshops, facilitated by one member from the LEAP and the PPI facilitator based in a mental health charity, were recorded within the MS Teams function with permission from all participants and transcribed. This method requires careful planning to build a safe space and open acknowledgement that information shared is not confidential and anonymous, with authors potentially identifiable to each other as well as those reading any output. Workshops made clear how data would be used, and the paper draft has been approved by all authors.

Workshops began with actor mapping. An example and template were shared with participants by a facilitator, but with the encouragement to use their perspective to shape their own version of the map. Workshop attendees could work on them after the session, as well as send in completed drawings when ready. Not all participants chose to complete the actor map; one follow‐up email was sent as a reminder. The facilitators were careful to plan sessions that were accessible and encouraged people to write in the ‘chat’ as well as speak in the session. The dynamic in workshops was helped by participants' familiarity with each other, including in the mixed group, where power differentials were named and openly spoken about.

### 2.4 Analysis Process

Transcripts were reviewed, and data were initially coded inductively both separately and together by the two facilitators using the six stages of Braun and Clarke's thematic analysis approach [35]. The positionality of the lead authors as lived experience researchers contributed to the active process of knowledge production in the initial construction of the themes [36]; which were then validated with the full LEAP and individual study team members. Data were analysed iteratively to generate themes, which were further refined throughout manuscript preparation. The writing process took several months and involved six drafts before reaching this version.

### 2.5 Payment

We adopted the payment system we had used throughout the study. LEAP members were paid £25 per hour for attending workshops and contributing to the paper. Study staff were remunerated through their institutions with salaries paid directly.

## 3 Results

### 3.1 Conceptualising Study Roles and Structures

We wanted to capture impact perspectives pictorially by asking those attending workshops to fill in an ‘actor map’. This reflected ‘who was who’ in the study (people, organisations and research infrastructure), their connections and linkages, and their relative perceived importance. We found strong consistency within the core study team maps and the LEAP maps, although they were comparatively different (see Figure 1). The LEAP lens primarily reflected their own role and structure, thus, working connections were perceived as strongest with the charity partner coordinating the LEAP. As one member explained:

So I would say that in this study, we've kind of sat outside this study team a little bit and we've kind of influenced via the PPI co‐ordiantor voice. She has acted almost as a conduit.

LEAP#4

**Figure 1:**
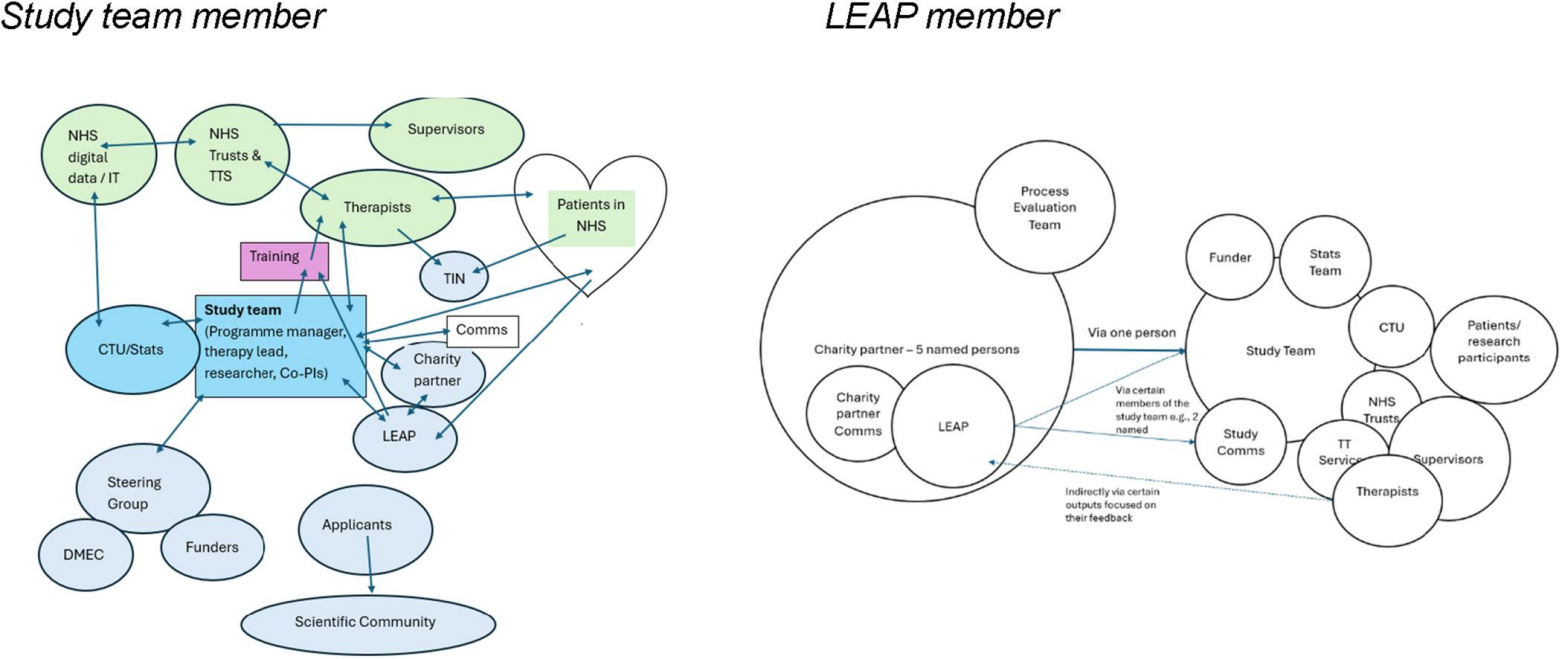
Diagrams outlining components of the study structure.

The study team had many more core connections and responsibilities; thus, maps were more complex with multiple actors having significant roles of equal importance. In workshop conversations, the study team placed emphasis on the therapists explaining:

For me we can't have done it without therapists in the project so they will be massive in the middle.

Team#1

Collaboration with public partners during the study often took place with individual study team members, rather than the whole team, creating individual relationships and working connections, rather than an inclusive research team culture.

An RCT within NHS talking therapies encompasses such a vast number of teams and people—I wonder if exposure to those wider teams might have helped with feelings of being more embedded (for the LEAP).

Charity #1

A key staff change occurred when the PPI co‐ordinator role moved from a university employee to the charity partner. This shifted the PPI model from embedded and working in the same office as other team members to outsourced and located separately, linked through regular communication in on‐line team meetings. We heard about ‘grumbles’ with the new PPI model feeling ‘divorced’ from core structures. It put pressure on key staff to work hard to establish trusting relationships.

The charity leading PPI being divorced again from the CTU and research team structure has been something that was quite unusual for me. And a bit unhelpful in you know, where there are sort of grumbles. Let's say you get the feedback and you disagree with it. It's almost easy to write off as saying oh that. Well, the LEAP. I don't even know who they are or their names.

Team#4

The same staff member also noted the benefits of this way of working and acknowledged, ‘I think we underestimate a lot in PPI work’. We learnt that managing expectations across the research team and LEAP was crucial, including when staffing responsibilities changed.

So many people came and went. So many changes. But every time that I attended the meeting, I always came out of a feeling that I had something to contribute and also I felt people felt valued and felt useful. And even with all the changes, it seems like every person that took part had kind of the same mindset. A willingness to help. This was my first experience doing patient involvement. I enjoy it very much.

LEAP#1

This required clear communication by listening to different perspectives and by taking time to ‘walk a mile in someone's shoes’. Ensuring LEAP meetings had space to help everyone connect person‐to‐person was vital.

A whole couple of hours chatting with people, walking around, talking about things … broke the ice a bit. And it was like it was a collaborative thing. You know, we work together and that data went into one of the worksheets.

TEAM#1

We also acknowledged the challenges and put effort into counterbalancing them to improve processes.

I would say some of the most impactful moments for me, when all of the complex moving parts really ‘clicked into place’ were less formal—an impromptu debrief with study team or LEAP members after a meeting, or meeting in person and discussing wider questions around the study during breaks.

Charity #1

This activity with the actor maps, helped surface how differently the study structures were perceived by staff members and the LEAP, and provided context for the different experiences they went on to explore.

### 3.2 Section Methods, 2: The Perceived Impacts of Lived Experience Involvement Across 7 Years

We identified two different ways in which lived experience involvement was perceived to shape and drive impact through the cycle of study creation, project implementation and research outcomes. These two elements of impact were: tangible and practical forms of influence, which are traditionally undertaken in research, such as contributing to intervention development or recruitment materials; and relational connections within wider academic and clinical systems, between multiple teams and the resulting impact on our ways of working. These two elements are reflected in both the perspectives of the university‐based researchers and lived experience partners.

We originally intended to explore PPI impact on research activities and study outcomes, however as we analysed workshop discussions, we found the data were broadly telling the story of research team culture. A team culture can either facilitate or hinder people feeling safe; it can also change after being nurtured at different times with new staff or strategies. This process highlights the symbiotic relationship between tangible impacts and relational connections and was described by various people in workshops as a ‘journey’. We identify a story‐arc that follows a trajectory of positive engagement and optimism, a period where things ‘got a bit choppy’ and there was ‘friction’, a penultimate phase was characterised by ‘just getting on with things’ and finally endings were managed through open reflection, including the writing of this paper. We identify key features of this journey (see Table 1) that explain why these four stages were communicated.

**Table 1:** Factors influencing our journey.

Components	Study team perspective	Public partner perspective	Impacts
(1) The study was founded with a co‐applicant team commitment to the value of LEAP involvement, integrating **lived experience expertise into the research**, which was sustained by the research team throughout the 7‐year project.	There was a strong commitment to lived experience perspectives right from the start by the leading academics behind the work. The LEAP ‘validated’ study team members’ commitment to undertake the project, as their ideas were supported, so they applied for funding with more confidence. Throughout, key research team members supported involvement activities and worked with the PPI coordinator to plan and communicate study progress.	The lived experience partners recognised how their experiences were genuinely valued by some study team members throughout high points, such as the process evaluation and science festivals. Such events were tangible and practical examples of impact. The LEAP identified moments when they felt their experiences were devalued. It was very hard to get some study team members to engage appropriately with the LEAP, with availability to meet low and interest mixed. The study ending, in writing this paper, was unifying and provided positive closure.	Overall, the sustained commitment to LEAP involvement and the value of lived experience from all members—university‐based staff, charity partner and the LEAP—meant that ‘choppy’ periods and ‘friction’ had reduced impact by the end. However, there were different perspectives on show in the data in how this ‘value’ was understood in practice. Relational connections with individual team members influenced the ways of working across the study. Ultimately, there could have been more impact from PPI if it had been more embedded throughout the study.
(2) **Levels of experience and confidence for embedding PPI** in a complex intervention trial design over 7 years.	For most study team members engaging a LEAP was a new way of working. There was strong support but less experience of how to do PPI in practice, and over time, some members did not meet with the LEAP at all. This was never acknowledged openly until we started this paper.	LEAP members varied in their levels of research involvement experience. The charity partner was a specialist involvement organisation with the PPI lead an expert in this field. Work with the LEAP was left to 3 key study team members by the end.	Over time, lack of confidence lessened as key members of the study team and LEAP became more familiar with PPI practices and each other. We were able to establish better ways to work together. However, PPI was never truly embraced by all—and several core team members were not involved with the LEAP.
(3) Who attended LEAP meetings and **mechanisms for study communication**.	In the beginning, study team members were attending LEAP meetings but tried not to outnumber members. This limited who could attend. Over time, less study team members attended, and this became the norm. PPI coordinator had a key role in communicating study progress to LEAP. Some study team would have preferred more contact with LEAP feeling ‘excluded’ but never vocalised this openly until this paper.	LEAP would have liked more contact with team members, and the study leads throughout. Members felt out of the loop during some periods but acknowledged role of both PPI coordinators in supporting the process. The PPI coordinator was a bridge attending study team weekly meetings and sending email updates to LEAP. Staff were encouraged by the co‐ordinator to attend LEAP meetings but they declined. The PPI coordinator wanted more proactive planning of LEAP involvement and better integration, but this was never achieved.	The relational connections were key to the ways of working adopted in the study and more cohesive team‐based relationships could have significantly improved ways of working. Inconsistencies had direct impact on team dynamics with LEAP feeling separate from university team for large parts of this study. This was in part due to Covid‐19. University team also felt distanced from the LEAP with core members having very limited contact for long periods. The PPI co‐ordinator was key but was under significant pressure to be a ‘bridge’ to individuals with different needs and prioritises.
(4) Expectations on what type of lived experiences were needed and **how to facilitate lived experience input into a mental health study**	Concerns that the original LEAP did not contain ‘relevant expertise’ and some members were contributing less well within first 2 years, led to a study team review of PPI. Also helpfully led to the setting up of a Therapy Involvement Network (TIN) group with participants from the trial recruited. Later these members joined the LEAP with one former trial participant contributing to the end of the study.	Very confident LEAP contained appropriate lived experience expertise for a study on common mental health problems with psychotic experiences from the beginning. Set up tried and tested PPI processes from the outset to deliver tangible ad practical forms of impact. However, felt the study team led PPI review which was not co‐produced, hindered trust building and working connections. Felt study team did not get to know LEAP members and there was an expectation to overly disclose.	Different viewpoints on what counted as relevant lived experiences, and assumptions about people's health experiences, significantly impacted several members of LEAP and the charity partner. These differences and not working through them early on made working together harder. It may explain why PPI was not truly embedded in this study.

### 3.3 The Value of Lived Experience: Both Tangible and Relational Impacts

The study was founded on a commitment to lived experience. The origin was validated by the LEAP, expressing support for a study addressing the needs of an underserved population. The study had purpose, and the results mattered to people with lived experience because they focused on a problematic gap in service provision. The study team reported how hearing this feedback from people with personal experience of psychotic experiences was instrumental. The co‐PI noted:

So in a way, the lived experience was the advocate for the disruptive ideas that we were playing around with… There were two psychiatrists going into a clinical psychology led NHS talking therapy service. It was nice to go in there with people with experience rather than alone as I think that puts us in a much stronger position.

(Team#2)

Moreover, the importance of this topic ‘resonated with my lived experience’ (LEAP#1), indicating its ongoing significance that also connected with study team members' own values and interests. A staff member talked about the project being ‘very personal’ (TEAM#1) based on experiences of working in services and understanding the need for new therapeutic approaches: ‘I felt very passionate about it. It was very important to me’ (TEAM#1).

The alignment of lived experience with clinical experience drove this study from project idea to funding application. The intervention was developed for individuals experiencing psychotic symptoms that were not considered severe enough for secondary care yet were not specifically addressed within primary care services delivered through NHS Talking Therapies (formerly IAPT). Described by one public partner as ‘Patients stuck in no man's land when accessing wellbeing services’ (LEAP#2), addressing this gap became a defining statement in the study's aim.

Lived experience also influenced the study in very tangible and practical ways, although there were'peaks and troughs’ (LEAP#2) of involvement. The project kept track using an impact log [37], which was compiled by the charity partner from meeting minutes and updated by core study team staff regularly. Examples included changes to terms used in recruitment materials and therapy training, systematic review criteria, input into newsletter copy, speaking at science festivals, reviewing results and working on the process evaluation study.

I'm not involved in doing the interviews. So you can feel like somewhat disconnected from the process and the people involved and then having that lived experience involvement makes you kind of, you know, brings it to life. And you're like, now I understand the importance of this and the real‐world impacts of the research. So I think there's also that personal impact that it has on you and your feeling towards the research as well.

TEAM#5

This is an established way in which lived experience is utilised to support research participants to understand and engage meaningfully with research, and reinforces confidence in the public that someone like them has shaped the design of research. The LEAP recommended using less‐stigmatising terms on participant recruitment leaflets, advocating the term ‘continuum’ of psychotic experiences to posit a normalising perspective on such symptoms. The importance was in:

Just making it more lay sounding and not gentle… That's the wrong word. But taking the stigma out of it and making it more approachable to patients.

(TEAM#1)

Lived experience involvement was also important because it helped the researchers to reflect ‘how was it gonna come across to a patient, you know’. (TEAM#1). LEAP members, however, felt some of their expertise was not always utilised effectively. They wanted more normalising language to be used in training manuals, whilst the study team opted for technical and psychologically focused terminology. As previously noted, relational connections within teams and ways of working were closely interlinked factors, one LEAP member felt disappointed that the input [from lived experience] ‘was kind of not listened to and not valued and kind of disregarded’. There was the belief that ‘This is how we do things in psychology and therapy… this is what's included in textbooks’ (LEAP#2). Such disappointment persisted during the study's lifetime and was reported by more than one LEAP member to impact relationships within the study. On reflection, one study team member noted: ‘It's a shame that we had a rocky, rocky start. Because, I think that has dictated quite a lot of the relationship throughout the programme. I think it could have been managed better at the beginning’ (TEAM#3). The PPI Co‐ordinator reflected:

The task of balancing clinical knowledge and preferences with loyalty to the reality of lived experience is not unique to this study. However, it is made even more complicated when a sense of good faith between those who prioritise clinical definitions and those who prioritise lived experience has been disrupted early on in the study. As co‐coordinator, I tried to mitigate this by communicating rationale (e.g. for language preferences) from both sides, and whilst there would be understanding, if the end result did not reflect a consensus, this would ultimately contribute to feelings of exclusion or incompatibility.

Charity #1

Debates about language and its importance cannot be underestimated; the choice of language used ultimately evidence which group holds the power in defining an experience and developing treatment [38].

### 3.4 Confidence in Doing PPI: Relational Connections and Ways of Working

In hindsight, we should have done more team‐building and shared learning early in the project (first 2 years), both during study design, including limitations and practicalities of intervention delivery in the NHS (what was possible and not). This would have enhanced relationships, forging better connections and effective ways of working at the start of the journey. At the beginning, team ethos became one of efficiency and project delivery to milestone:

… driven by milestones, by dates having this done and this obviously limited the amount of conversations or dialogue with others. That was an issue that we had to resolve because obviously efficiency sometimes brings the risk of not listening.

TEAM#2

Lack of overall confidence in doing PPI, and relationships that were still developing, contributed to some tensions over how lived experience contributions were perceived. Meaning that less visible methods of contribution were undervalued by the university research team. It was reported that such issues led to some members:

Sometimes resenting individuals in the LEAP coming in and not doing much and getting paid and that's set up as sort of dynamic that needed a bit of management within the team.

TEAM#2

A survey by the study team surfaced key issues which we all tried to address in the middle part of the study. It confirmed the work for some was ‘daunting’.

I think I wanted to get this right. You know, it wasn't that I didn't do it or didn't see the point of it was, how do we do it well? Make sure everybody is being able to contribute.

TEAM#1

### 3.5 Planning Meetings and Study Communications: Relational Connections Leading to Tangible Impact

The LEAP emphasised that high points of involvement were based on relational connections with individual study team members, as in the process evaluation or systematic literature search. Fostering a team culture is a key part of any study delivery and requires transparent and honest communication; misunderstandings can haunt a project. There were some misunderstandings, we learnt later, about how to manage meetings with the LEAP early in the project. Some study team members reported at one stage they were ‘discouraged’ from going to LEAP meetings because they received feedback that the LEAP were ‘overwhelmed’ and that *‘*made it this kind of them and us thing that was very uncomfortable’. A second person noted: ‘We almost felt a bit excluded’ (TEAM#2). On the contrary, many LEAP members felt they would have liked even more contact with a study team member, stating they needed ‘to have that opportunity of interacting with the study much more than it currently is’ (LEAP#2). The charity partner reflected on the reasons for these different viewpoints as it was core to their role to manage communication between the LEAP and study team from mid‐way.

Balancing the needs of everyone in this study was impossible. Early team dynamics were hard to navigate. Looking back we should have done more reflective work and addressed the problems more directly but no‐one, including us, was ready to do that at the time.

Charity #1

Methods of lived experience involvement varied, with the opportunity to contribute to knowledge generation in flexible ways. This was impacted by COVID and a move to remote meetings. Power plays a significant role in teams and is essential in building team cohesion on a study journey. At the start of the study, when face‐to‐face meetings dominated, one LEAP member noted:

They [study team] all sat separately and we all sat separately and it just happened naturally and you could, you could feel the power dynamics in the room.

LEAP#2

However, by the study's end, one LEAP member commented that respect for lived experience was felt across the whole research team.

And it's helped me to understand that like you don't have to be completely, you know like academic to be part of this study, your lived experience counts equally, so that's been important for me.

LEAP#3

To facilitate effective involvement, it is important to have a flexible approach to ways of working to enable people with lived experience to contribute; this is essential to team cohesion both within LEAP and in their relations with other groups. This was commended by one LEAP member ‘when I wasn't capable enough to contribute I felt that people drew out what they needed’. She continued:

We're always so encouraged to say [what we want] and reassured that it's OK if you don't.

LEAP#1

It was important to recognise and celebrate when ‘impact’ ‘benefitted the study; particularly when tensions and misunderstandings were visible’. The two‐way nature of this ambition was described by the PPI co‐ordinator:

Where we did create feedback loops—such as hearing from the therapists or study team members how much a LEAP member perspective can help bring the intervention to life in ‘real’ terms, or the benefits to the LEAP of being involved in the study—that's where I really felt you could see the impact of PPI, how it changed or enhanced people's professional and personal development pathways.

Charity #1

### 3.6 Dealing With Tensions When Expectations Diverge: Managing the Impact of Relational Connections

A lack of team cohesion was experienced across the study journey at key points. It was most significant in the first 2 years. Divergent views emerged, prompted by questions about the types of lived experience that should be represented in the LEAP.

The research team felt that we didn't have the right lived experience actually supporting us.

TEAM#2

The study's focus was working with an under‐described group whose needs are often not served within talking therapy services. The LEAP had personal knowledge of psychotic experiences through their own mental health journey, including treatment in IAPT services (now called NHS Talking Therapies), but were not supported under the same four service structures as the participants in the study trial. Hearing challenges about their perceived expertise was hard for LEAP members.

I also felt like my own lived experience was like, I was basically told, oh yeah, well, I was basically told that my own lived experience wasn't relevant. That then did leave a bad taste of my mouth.

LEAP#2

At the beginning, when the LEAP was bigger there were also expectations on how members should contribute, with preference from academics for dialogue in meetings. However, some LEAP members needed time to process information and provided feedback outside of sessions making their contributions less visible to the study team. These practices fed assumptions, by academics, about appropriate relevant lived experience among the group.

To address the need for lived experience of the actual intervention being delivered in the trial a second group of lived experience advisors was recruited part way through the study (around Year 4)—Therapy Involvement Network (TIN)—consisting of people who had received the study intervention and therapists delivering the intervention. In the last 2 years of the project, the TIN members with lived experience were integrated into the overall LEAP group. These members contributed to sense‐making of findings and recommendations for next steps, bringing different ‘relevant’ lived experience into the conversation. This combination worked well for some of the academics; ‘I'm glad that we had such a broad group’. #TEAM4.

The dilemmas within this study about what constitutes relevant lived experience and how it should be represented in research are rooted at the heart of all PPI and impact all levels of team culture. The co‐PI reflected on the ‘more conceptual question about what lived experience means particularly in mental health research’ noting that many mental health researchers have lived experience, and they can find it ‘quite stigmatising and siloing when they're told that they don't have any lived experience’. The ‘idea of wearing two hats’ and the notion of a hybrid academic and lived experience identity are difficult to negotiate both in the mental health research world and in academic teaching and learning [39]. Debates about who possesses the ‘right’ or ‘relevant’ lived experience are regularly expressed when lived experience educators contribute to the related fields of health and social care education. Does the person have the relevant skills to teach? Has regular involvement led to them becoming too professionalised to convey authentic experience [40]? Such challenges, endemic in academia, are particularly difficult in a study team who have low collective confidence, trust and experience in doing PPI. The direct impact of this dilemma was experienced in this study when a person with lived experience located in the academic team changed roles in the study team. A team member reflected on this situation:

You know, you're sort of not believed in the new role. You're not as legitimate as you were in the old role. It's as much about how an individual is given much of their identity by other people as well. It's the identity that they portray and the identity they're given.

TEAM#2

Power differentials within academic research teams can impact on the different values placed on the various forms of knowledge and expertise. LEAP members, however, valued the diversity of their group and different abilities and skills:

Having fellow LEAP members who are so knowledgeable is so valuable but also people who have suffered is extremely valuable especially with struggles with English as a second language and tech. Never made to feel small or lower.

LEAP#3

This shared respect led to a distinct group identity in which the LEAP became a ‘safe space’. This emphasised team cohesion and shared authenticity.

I think my experience among us as LEAP members has been good because we've been able to support each other and be able to see each other for such a long time and come to like you know, we have the commonality of being LEAP. So it's been a safe space. It's helped me grow.

LEAP#3

In another example, one LEAP member provided their description of the experience as ‘very precious’

So I feel it is very very precious that I was able to participate in this group and able to last for so long. And the impact in my life. I think just being exposed to something that I had never been exposed before and see that my lived experience was or is worth it like it's it's rich.

LEAP#1

## 4 Discussion

This paper has sought to uncover the different and shared perspectives of PPI impact within an NIHR‐funded complex mental health intervention research study over 7 years, among the study team and LEAP members. This is an honest account, and a difficult one, which is rarely surfaced in academic literature.

### 4.1 First Author Reflections

As a LEAP member and the charity facilitator, we were given the privilege of collecting and making sense of the data. We spent a long‐time planning how to run the workshops and checked in with the study team when developing our protocol, submitting for research ethics with the co‐PI, reviewing key themes and drafting this paper. Our approach was, however, founded on the positionality of our roles, and this requires open acknowledgement and reflection. The LEAP member is a university‐based and lived experience researcher whose career has developed during the study lifetime. Study peaks have been a source of personal growth, and the troughs a source of frustration. As reflective of the wider study structure and mentioned in this paper, the charity worker's position as co‐ordinator meant they were tasked with the challenge of negotiating the diverse expectations of all study partners. The process of manuscript preparation between all study partners has been cathartic and healing, allowing a sense of collaborative learning, which was at times missing during the study.

We feel there was a genuine commitment to PPI, but a concern about how to do it properly. There was at times distance and a lack of transparent communication, and misunderstandings such as who should attend meetings and why. There were genuine examples of excellent practice with individuals, including the process evaluation, based on shared values and respect. Sustained effort was made to incorporate the lived experience into the study right until the end, but the questioning of people's personal, direct lived experiences was an ever‐present reminder of whose view counted most and haunted the project. The emotional labour [41] of lived experience work is increased when differences of opinion are not addressed constructively, but working through them can be a source of innovation [31].

While there were divergences in perspectives and experiences shared in this paper, there was also a unified sense of commitment to study aims. As a co‐ordinator and someone with my own lived experience, I took on the task and privilege of attempting to gather reflections from colleagues with a range of roles and positions involved in the study. It is a constant balancing act, which requires a great deal of confidence, especially when navigating long‐ingrained professional hierarchies, acting as an impartial conduit, and knowing when to provide my own perspective.

Because of our belief in the importance of providing an analysis of impact that goes deeper than quantitative measures, we also had to commit to sharing our experiences in a way which was candid, transparent, and reflective. It resulted in a process which raised similar challenges to that of PPI in an RCT—how do we achieve the most flexibility and collaboration within the confines of the research framework? How are personal experiences mitigated through the process of research and paper writing? How is trust cultivated and maintained to enable honest reflections? Who gets to tell whose story? And how do we negotiate the inherent power of authorship whilst ensuring that everyone feels as though their perspectives have been fairly reflected? These are the challenges we have tried to address both through researching for this paper and in the act of writing it.

### 4.2 The Peaks and Troughs of Involvement

The project was founded in a commitment to lived experience involvement, and a passion and intent to support the needs of an under‐served population by the study's clinical academics. Our conversations surfaced stakeholder experiences explained in part by varied levels of experience and confidence in doing PPI, lack of trust in recruitment of relevant lived experience to the study, COVID and moves to remote meetings impacting research team dynamics, and a multi‐site complex study with different teams spread across the United Kingdom and beyond. The actor maps reveal how we experienced the study in different ways, depending on our role and day‐to‐day contact with key elements of delivery, including therapists and supervisors, the CTU, partner recruitment sites and the charity partner. This led to feedback on impact that focused more on the journey of our study rather than individual activities where lived experience influenced a study decision or output. There were many tangible and practical examples of impact, but these were built on relational connections which influenced the different ways of working. We understood a story‐arc that followed a trajectory of positive engagement and optimism, a difficult period with friction and tension, a core period of just getting on with things and a final phase ending positively with collective learning and reflection.

### 4.3 Whose Lived Experience Counts?

A key challenge in this work was the conversation about lived experience on two levels. First, some study members felt they had the ‘wrong’ people in the LEAP, which left individuals with relevant lived experience to the study aims feeling angry and invalidated. Second, the difficulty of being both a member of an academic research team and a person with mental health experiences was met within an environment unfamiliar to working with lived experience researchers. This is not a new phenomenon—others have written about the same challenges within mental health services [13, 42], mental health research [43] and in higher education settings [19]; indeed, academics often report a reluctance to share their own lived experiences due to both perceived and actual stigmatisation of being identified as being less capable [44].

### 4.4 Reflections on ‘Measuring Impact’

The initial purpose of this article was to understand the impact of lived experience involvement in a mental health project that had received significant resource investment from the NIHR. Trying to measure or assess PPI in this way has been referred to as ‘forcing a square peg into a round hole’ [26]. We found that public involvement in research is evolutionary, unpredictable, subjective and context‐specific, requiring conversations to unpick learning. At the heart of PPI are relationships and the quality of interactions that enable critical conversations and radical candour, also known as the position of a ‘critical friend’ [6]. There are also calls for a critical research agenda for PPI that ‘[a] considers public involvement not as an instrumental intervention but a social practice of dialogue and learning between researchers and the public; [b] explores how power relations play out in the context of public involvement in health research, what empowerment means and whose interests are served by it, and [c] asks questions about possible harms as well as benefits of public involvement, and whether the language of impact is helpful or not’ [45]. This encompasses not only tangible and practical impact, but also the impact of relational connections, which inform ways of working and team culture.

Mockford et al. [21] highlight the need to quantify the value of lived experience, however, our data presents a story of building connections over time and distance. Based on interpersonal and interprofessional relationships, impacted by issues including power linked to role status and the dominance of the bio‐medical model in mental health care and research. There is a critical distinction to be drawn between measuring impact and evaluating contributions. The former process is often technocratic, instrumental, largely quantitative, and summative, whilst the latter activity might be reflexive, imaginative, and collaborative. Therefore, we would highlight the need to have ongoing conversations throughout the research process to reflect on and share ideas—to improve and enable team collaboration. Martin et al. [46] reinforce the importance of collaborative relationships across a whole project team of academics and people with lived experience, built on foundations of respect and value. Richmond et al. [24] underline the need to develop supportive relationships with public contributors, offering accessible ways to engage. Thus, it is necessary for transparent and ongoing communication between all parts of a study, not only with the LEAP, to build and sustain involvement. Ongoing reflection and honest sharing of ideas and opinions may have avoided some of the misunderstandings in our study and may have allowed us to celebrate the successes. Thus, full contribution and participation need to be advocated and nurtured across a study's lifetime.

From our experiences, each project builds unique ways of involving and supporting public partner contributions. Overall, this article reveals that just as PPI impact is a nuanced concept, as is the learning journey of all studies. Our data reveals a journey of sustained effort and intent by members of a team, with a shared commitment to improve service delivery for an under‐served population. Careful, considerate teamworking, reflection and collaboration support the development of PPI partnerships that are invaluable and impactful.

So I think all parties had quite a journey to go on before we could have a full meeting of minds, however it's added to my learning that inclusion, lived experienced inclusion like this, however difficult it is, it's always better if you work it through. The research is always better.

#team2

### 4.5 Limitations

It is important to acknowledge that our study only includes the views and experiences of a small number of people who consented to reflect together—6 staff members and 7 persons connected to the LEAP. The narratives of other people involved may have been different. However, our sample did contain the core team and those members who had been with the study longest; of the 13 participants, 10 were in post at the start of this journey.

This paper follows a qualitative case study methodology, but was only conceptualised towards the end of the 7‐year programme period. It would have been beneficial to have explored the impact reflectively throughout, building a larger data set collected across several time points. There may be retrospective, recall and selection bias as well as constrained narratives because of group dynamics in workshops with senior academics alongside more junior staff. Moreover, the positionality of the first two co‐authors as lived experience researchers may have impacted initial analysis of data as discussed earlier, but validation with other team members provided a counter‐balance to potential interpretation bias. Our work is context‐specific, utilising a case study design, which may limit its wider application to other settings, but we hope it is useful to other mental health research study teams.

## 5 Conclusion

Impact is about much more than a quantifiable value. It is a nuanced term that benefits from multi‐perspective explorations to move beyond surface‐level observations to achieve a deeper understanding of how lived experience expertise shapes complex research processes across extended time periods, including the role of team dynamics, built on relational connections. Working with lived experience partners can add value to a study. We saw that in validating the study concept, working within the process evaluation and supporting trial set up, including intervention training design. It does, however, require an openness to changing team culture and benefits from regular reflective learning with open feedback.

## Author Contributions

**Joanna Fox:** conceptualisation, investigation, writing–original draft, methodology, writing–review and editing, formal analysis. **Roya Kamvar:** conceptualisation, investigation, writing–original draft, methodology, writing–review and editing, formal analysis, project administration. **Vanessa Pinfold:** methodology, conceptualisation, writing–original draft, funding acquisition, writing–review and editing, supervision. **Annabel Walsh:** writing–review and editing, investigation. **Polly‐Anna Ashford:** investigation, writing–review and editing, funding acquisition. **Rosie Lindsay:** investigation, writing–review and editing. **Jesus Perez:** investigation, writing–review and editing, funding acquisition. **Peter B. Jones:** conceptualisation, funding acquisition, writing–review and editing, investigation. **Debra Russo:** investigation, writing–review and editing.

## Ethics Statement

This work was approved by Cambridge Psychology Research Ethics Committee on 24/3/2024—PRE.2025.004

## Consent

The authors have nothing to report.

## Conflicts of Interest

The authors declare no conflicts of interest.

## Permission to Reproduce Material From Other Sources

The authors have nothing to report.

## GRIPP2 Short Form

**Table:** 

Section and topic	Item	Reported on page no
1: Aim	Describe the purpose of PPI in the study.	Page 4
2: Methods	Explain how patients and the public were involved	Pages 4–6
3: Study results	Report the outcomes of PPI, including positive and negative effects.	Pages 7–17
4: Discussion and conclusions	Comment on the influence of PPI on the study overall.	Pages 18–21
5: Reflections/critical perspective	Provide insights, lessons learned, and any challenges encountered	Page 18

## Data Availability

Data are available from the authors on request.
